# Glycogen Storage Disorder Type IXb: Exploring Clinical Patterns and Genetic Insights Into a Rare Phosphorylase Kinase B (PHKB)-Associated Case

**DOI:** 10.7759/cureus.65474

**Published:** 2024-07-26

**Authors:** Inuganti Venkata Renuka, Sudhakar Ramamoorthy, Vijayalakshmi B, Srilaxmi N, Bakkamanthala S K Kanth

**Affiliations:** 1 Pathology, NRI Medical College, Chinakakani, IND; 2 Pediatrics, NRI Medical College, Chinakakani, IND

**Keywords:** whole-exome sequencing, gsd ix, hepatomegaly, phosphorylase kinase b, glycogen storage disorder

## Abstract

Glycogen storage disorders (GSDs) encompass a group of metabolic disorders resulting from deficiencies in enzymes involved in glycogen synthesis or breakdown. Among these, GSD type IX manifests due to a deficiency in phosphorylase kinase enzyme, leading to liver-specific, muscle-specific, or combined forms of the disorder. We present a case report of an exceedingly rare deletion-type mutation in the phosphorylase kinase B (PHKB) gene causing GSD type IXb, offering a comprehensive evaluation of clinical, laboratory, and molecular findings. A one-year and four-month-old male, born of third-degree consanguinity, presented with delayed motor milestones, hypotonicity, short stature, doll-like facies, and hepatosplenomegaly. Preliminary investigations revealed fasting hypoglycemia, ketonuria, elevated liver enzymes, and histological evidence of glycogen accumulation. Whole exome sequencing identified a homozygous deletion encompassing exons 2 to 10 of the PHKB gene, confirming the diagnosis of GSD IXb. GSD IXb due to PHKB mutations is rare, comprising only 10% of liver-specific GSD IX cases. Compared with similar cases reported in the literature, our analysis highlights the genetic heterogeneity within this subtype. Although clinical manifestations may overlap, specific genetic alterations vary, indicating that an individualized diagnostic approach is needed.

## Introduction

Glycogen storage disorders (GSDs) stem from enzyme deficiencies in glycogen synthesis or breakdown and are categorized into liver, skeletal muscle, and combined types. GSD type IX results from a phosphorylase kinase enzyme deficiency, with subtypes determined by specific gene mutations [1]. Phosphorylase kinase enzyme has four subunits namely, α, β, γ, and δ. The α subunit of the enzyme is encoded by two genes, PHKA1 and PHKA2, the β subunit by the phosphorylase kinase B (PHKB) gene and the γ subunit by the PHKG2 gene. Mutations or pathogenic variants in PHKA1 cause X-linked muscle-specific GSD IX, PHKA2 leads to X-linked liver-specific GSD IX, PHKG2 causes autosomal recessive liver-specific GSD IX, and PHKB results in autosomal recessive GSD IX (GSD IXb) affecting both liver and muscle. The calmodulin gene encoded the δ subunit and is not associated with any GSD. The estimated prevalence of GSD IX among the general population is 1 in 100,000, contributing to 25% of all GSD types. Among GSD IX types, GSD IXa is the most common and accounts for 75% of the cases. GSD IXb and GSD IXd types are rare, of which the former contributes to 10% of GSD IX cases and the prevalence of the latter is unknown. Patients with liver-specific GSD IX present with hepatomegaly, growth restriction, ketosis, and hypoglycemia; however, muscle-specific GSD IX patients present with exercise intolerance and muscle weakness. PHKB mutated GSD IXb often show prominent liver findings with subtle or no muscle symptoms thus mimicking liver-specific GSD IX subtypes, PHKA2 and PHKG2. Due to these overlapping presentations and differences in treatment and surveillance guidelines across GSD IX subtypes, identifying the subtype by genetic testing becomes crucial [2]. This case report outlines an exceptionally rare deletion-type mutation in the PHKB gene causing GSD type IXb and presents comprehensive clinical, laboratory, and molecular findings.

## Case presentation

**Figure 1:**
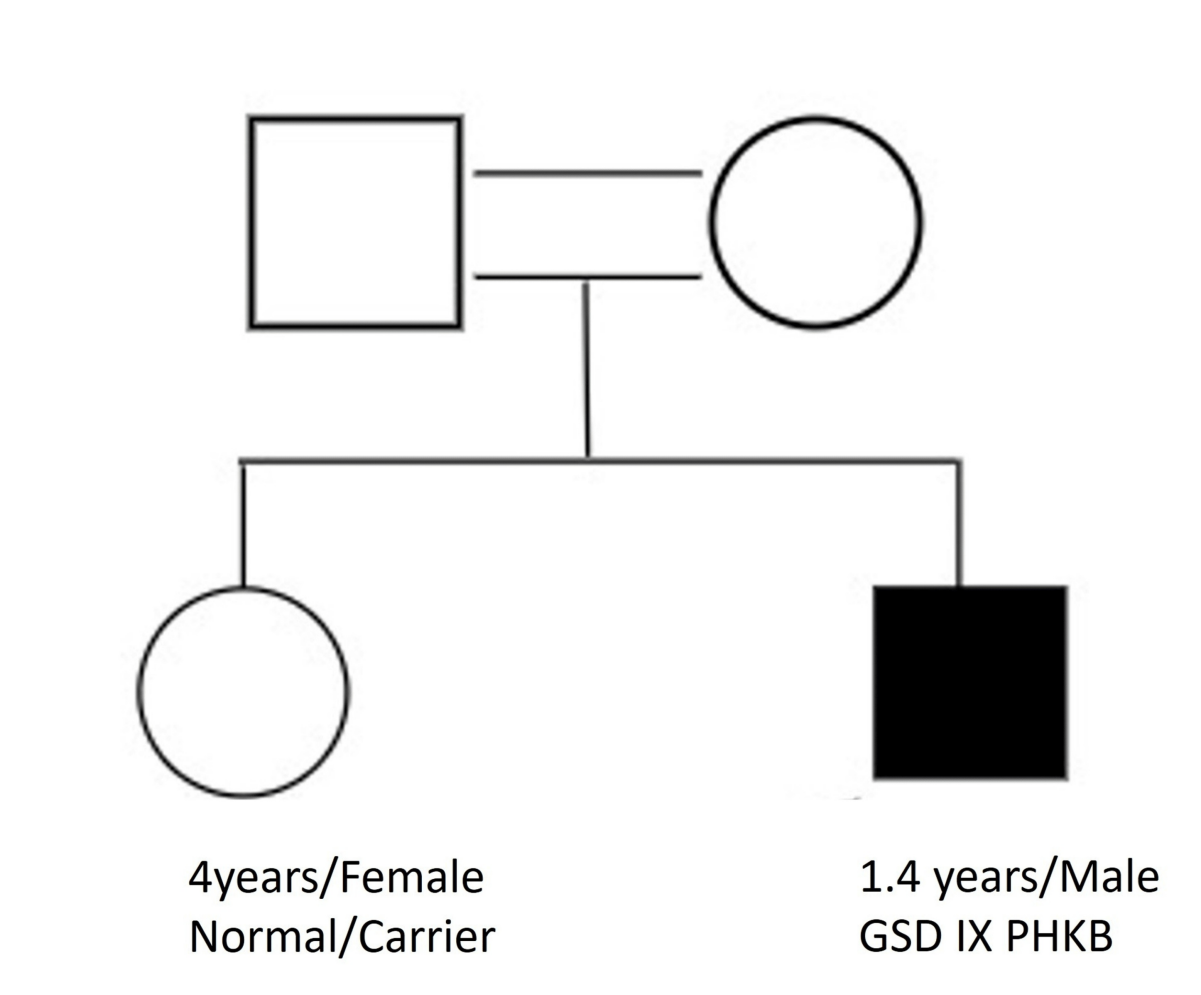
Family pedigree chart

A one-year and four-month-old male, born of third-degree consanguinity, presented with an inability to walk or stand even with support. The patient was a term baby with no visible deficit at birth. Later he developed short stature, doll-like facies, and moderate hepatosplenomegaly. Physical examination revealed delayed gross motor milestones and hypotonicity of both bilateral upper and lower limbs. The patient has an elder sister who was asymptomatic during the time of the investigation. Figure 1 illustrates the family pedigree chart of the proband.

Preliminary lab investigations revealed fasting hypoglycemia, ketonuria, elevated liver transaminases, hyperammonemia, and hypertriglyceridemia. In contrast to the patient’s muscle-specific symptoms, creatine kinase levels were not elevated (Table 1).

**Table 1:** Initial laboratory assessment on admission AST: Aspartate transaminase; ALT: alanine transaminase; ALP: alkaline phosphatase; HDL: high-density lipoprotein; LDL: low-density lipoprotein; APTT: activated partial thromboplastin time

Laboratory parameters	Value	Reference range
Fasting plasma glucose	57 mg/dl	74-106mg/dl
Urine ketones	Positive	Negative
Total bilirubin	0.6mg/dl	0.2 – 1.3 mg/dl
Direct bilirubin	0.3 mg/dl	0.02 – 0.2 mg/dl
Indirect bilirubin	0.3mg/dl	0.2 – 1.0 mg/dl
AST	129 U/L	17-59 U/L
ALT	77 U/L	15-60 U/L
ALP	347 U/L	38-126 U/L
Creatine kinase	37 U/L	55-170 U/L
Serum ammonia	219.20 umol/L	9-30 umol/L
Serum cholesterol	136 mg/dl	<200 mg/dl
Serum triglycerides	246 mg/dl	<150 mg/dl
HDL	16 mg/dl	40-60 mg/dl
LDL	71 mg/dl	<130 mg/dl
Prothrombin time	11.2sec (INR: 1.97)	12-14 sec
APTT	25.7 sec	22.2-30.7 sec
Lactate	0.61 mg/dl	4.5-20 mg/dl

**Figure 2:**
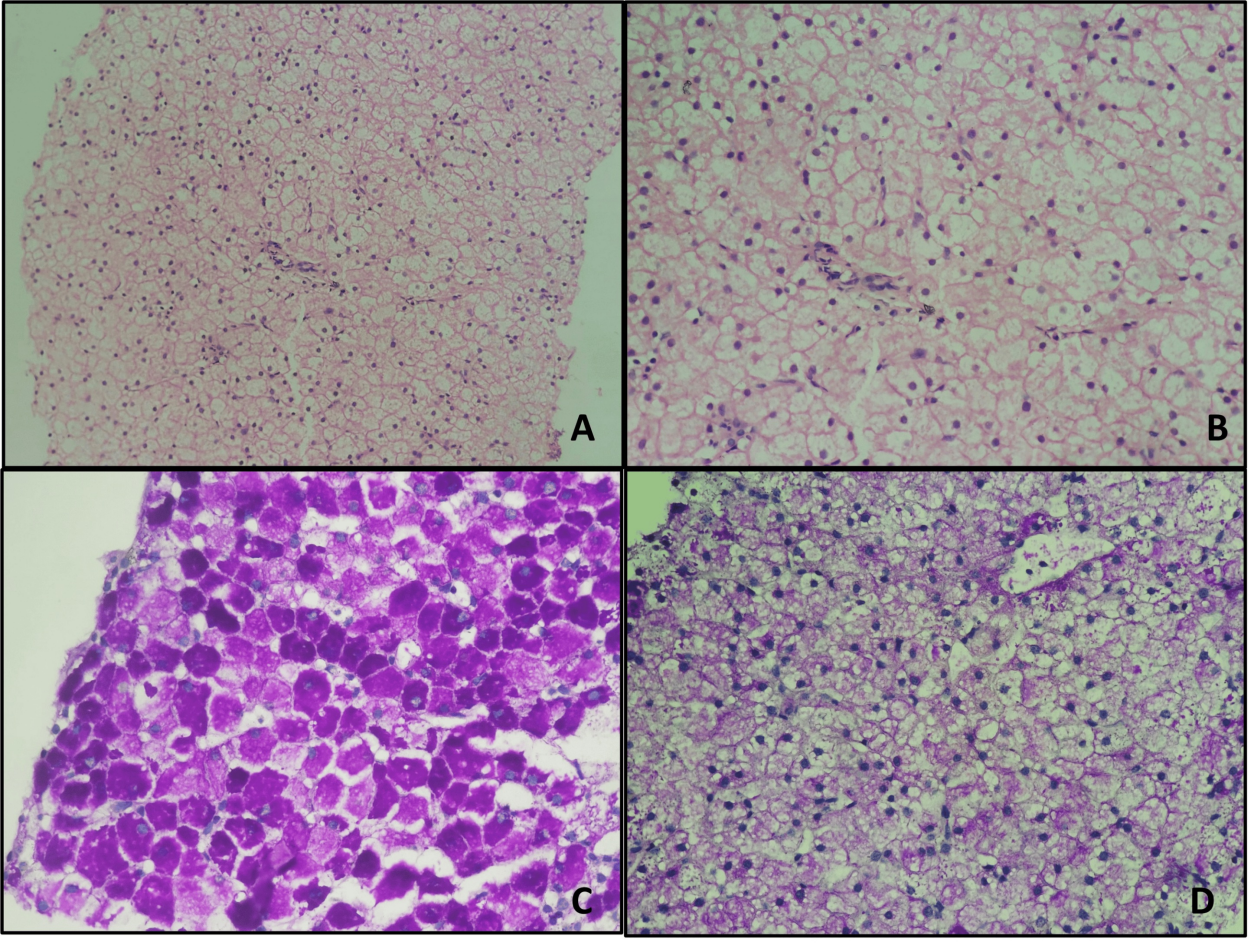
Histomorphology of the transcutaneous liver biopsy A. Overview at low magnification reveals maintained lobular architecture with an unremarkable portal tract(H&E, 100x). B. The hepatocytes are distended with well-defined cell boundaries and clear cytoplasm (H&E, 100x).  C. Periodic acid Schiff stain highlights the involved hepatocytes by imparting dense granular positivity with alternating zones of normal hepatocytes that are light stained, creating a mosaic pattern (PAS, 200x). D. The dense granular magenta positivity is digested by diastase resulting in clear cytoplasm (PAS-D, 200x).

A transcutaneous liver biopsy was performed, which, on microscopic examination, exhibited maintained lobular architecture with unremarkable portal tracts (Figure 2A). The hepatocytes were distended by tiny vacuoles, which alternated with near-normal-looking hepatocytes with pink granular cytoplasm, exhibiting a mosaic pattern (Figure 2B). Periodic acid Schiff stain showed strong intracytoplasmic granular positivity in hepatocytes (Figure 2C), which were digested by diastase (Figure 2D), thus confirming glycogen accumulation. Based on the clinical, biochemical, and morphological features, a diagnosis of GSD was rendered in the biopsy.

**Figure 3:**
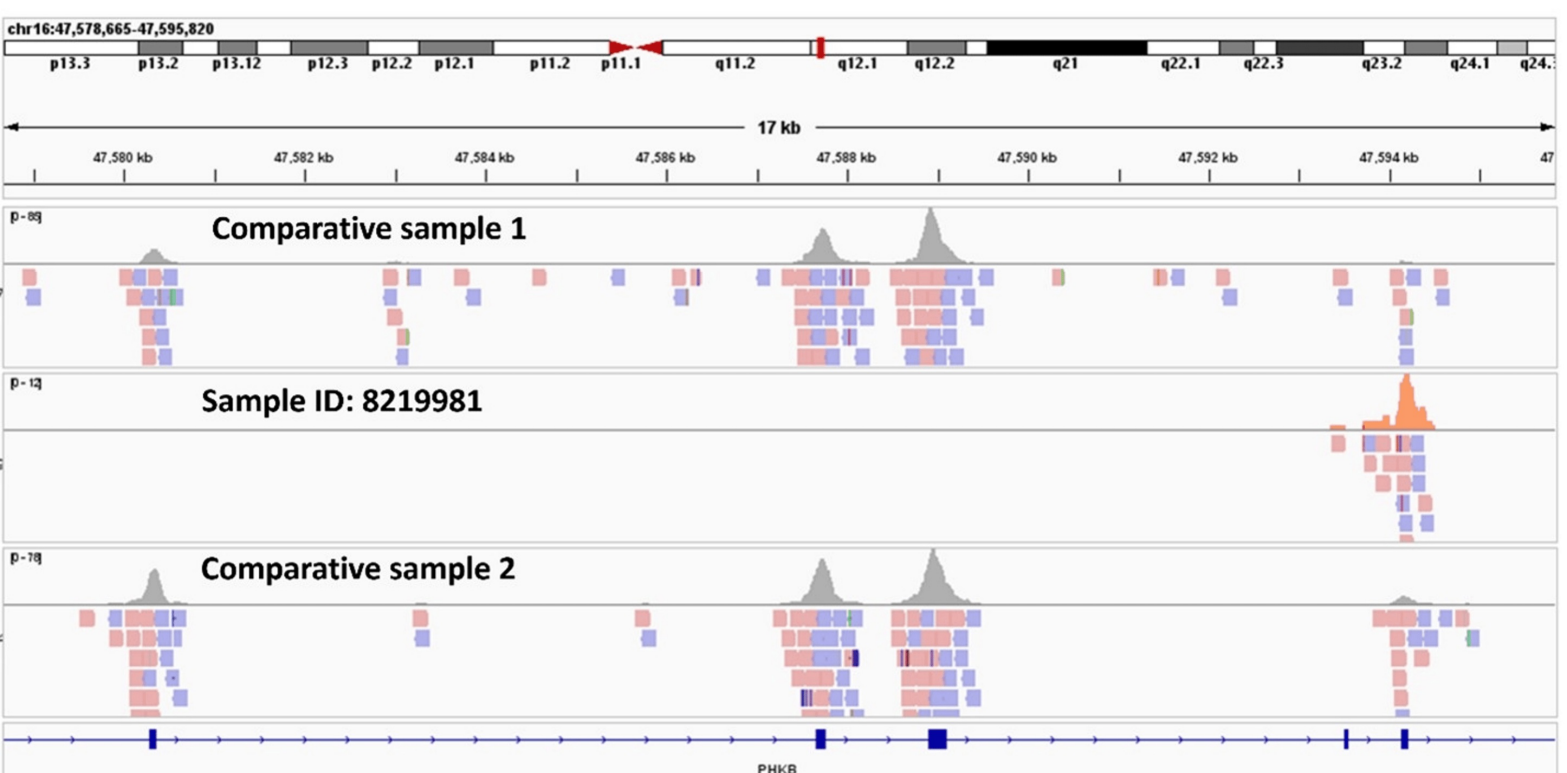
Integrative Genomics Viewer (IGV) image for the Chr 16 c.(76+1_77-1)_(1068+1_1069-1) deletion

The patient’s blood sample was sent for whole exome sequencing, which revealed an exonic homozygous deletion of size [~125.22KB], on chromosome 16 chr16:g.(47461427_47497398)_(47589103_47593499)del encompassing exons 2 to 10 of the PHKB gene [ENST00000323584.10; c.(76+1_77- 1)_(1068+1_1069-1)del] (Figure 3) (Table:2). The variant was classified as “likely pathogenic” to cause PHKB associated GSD IXb. As per the American College of Medical Genetics and Genomics (ACMG)/American Association of Molecular Pathology (AMP), both the terms “pathogenic” and “likely pathogenic” were considered diagnostic and can be utilized for clinical decision-making [3].

**Table 2:** Variant table

Gene	Phosphorylase kinase B (PHKB)
Location	Exons 2-10 of Chromosome 16
Variant	c. (76+1_77-1)_(1068+1_1069-1)del
Mutation type	Homozygous deletion
Disease	Phosphorylase kinase deficiency of liver and muscle (GSD IXb)
Inheritance	Autosomal recessive

Subsequently, a specific diet plan (protein-25%, carbohydrate-40% & fat 35% of total calorie intake, corn starch at night) was advised. The patient was referred to a higher center for genetic counseling and carrier testing of the patient’s sibling.

## Discussion

PHKB-mutated GSD IXb is rare attributing to only 10% of all liver-specific GSD IX. More than 20 pathologic variants of the PHKB gene have been reported so far in this subtype, of which only less than 10% results from deletion-type mutation [2]. A comprehensive analysis, as depicted in Table 3, juxtaposes our findings with three other cases of deletion-type mutation of PHKB-associated GSD IXb reported in the literature. This analysis encompasses age, clinical presentation, biochemical values, and histomorphology, drawing parallels among cases described by Burwinkel et al. [4,5] and Davit-Spraul et al. [6]. Our patient presented with a doll-like face, muscle weakness, and delayed motor milestones similar to the case reported by Burwinkel et al. [4].

**Table 3:** Patient characteristics and deletion-type PHKB mutations reported in the literature PHKB: Phosphorylase kinase B

	Current study	Burwinkel et al. [4]	Burwinkel et al. [5]	Davit-Spraul et al. [6]
Age/sex	1.4yrs/Male	2.1yrs/Male	4yrs/Male	NA/Female
Ethnicity	Indian	German	Israeli-Arab	Not available
Clinical features	Doll-like face, muscle weakness, delayed gross motor milestones	Asymptomatic	Doll-like face, abdominal distention, muscle weakness	Asymptomatic
Hepatomegaly	Yes	Yes	Yes	Yes
Lab values	Fasting hypoglycemia, high triglycerides, ketonuria, high liver transaminases	Hypoglycemia	Not available	Elevated transaminases
Liver biopsy	Distended hepatocytes with PAS-positive and diastase-sensitive swollen cytoplasm	Not performed	Not available	Not performed
Mutation in PHKB gene	c.(76+1_77-1) _(1068+1_1069-1)del	c.306-2 A>G	7574 bp deletion	c.573_577 del GATTA
Variant type	Deletion of exon 2 to 10	Deletion of exon 5	Deletion of exon 8	Deletion of exon 6

However, it is noteworthy that the specific exons affected by the deletion varied among the cases, indicating genetic heterogeneity within this subtype that didn’t reciprocate on clinical or pathological features. The identification of deletion-type mutation of PHKB involving exon 2 to exon 10 in our patient adds to the expanding spectrum of PHKB mutations associated with GSD IXb. Understanding the genotype-phenotype correlations and the molecular mechanisms underlying these mutations is crucial for elucidating the pathophysiology of this disorder and for the development of tailored therapeutic interventions. The primary goal in these patients is to maintain blood glucose levels between 70 and 100 mg/dl and to maintain beta-hydroxybutyrate between 0.0 and 0.2 mmol/L by dietary modifications customized to the patients. Follow-up surveillance of liver ultrasound every 12 to 24 months and echocardiogram approximately every two years are suggested to prevent long-term complications like liver cirrhosis and interventricular septal hypertrophy reported in PHKB mutated GSD IXb patients. An exercise program by a physiotherapist is suggested to alleviate muscle-related symptoms [2].

## Conclusions

This case underscores the rarity and diagnostic challenges associated with GSD type IXb, caused by a rare deletion-type mutation in the PHKB gene. By elucidating the clinical manifestations and genetic underpinnings of this disorder, we emphasized the crucial role of genetic testing in its accurate diagnosis. Knowledge of clinical presentations and laboratory parameters of this entity along with early diagnosis by genetic testing will prevent potential long-term complications.
